# Effects of Electrical and Structural Remodeling on Atrial Fibrillation Maintenance: A Simulation Study

**DOI:** 10.1371/journal.pcbi.1002390

**Published:** 2012-02-23

**Authors:** Trine Krogh-Madsen, Geoffrey W. Abbott, David J. Christini

**Affiliations:** 1 Greenberg Division of Cardiology, Department of Medicine, Weill Cornell Medical College, New York, New York, United States of America; 2 Institute for Computational Biomedicine, Weill Cornell Medical College, New York, New York, United States of America; 3 Department of Pharmacology, Weill Cornell Medical College, New York, New York, United States of America; University of California San Diego, United States of America

## Abstract

Atrial fibrillation, a common cardiac arrhythmia, often progresses unfavourably: in patients with long-term atrial fibrillation, fibrillatory episodes are typically of increased duration and frequency of occurrence relative to healthy controls. This is due to electrical, structural, and contractile remodeling processes. We investigated mechanisms of how electrical and structural remodeling contribute to perpetuation of simulated atrial fibrillation, using a mathematical model of the human atrial action potential incorporated into an anatomically realistic three-dimensional structural model of the human atria. Electrical and structural remodeling both shortened the atrial wavelength - electrical remodeling primarily through a decrease in action potential duration, while structural remodeling primarily slowed conduction. The decrease in wavelength correlates with an increase in the average duration of atrial fibrillation/flutter episodes. The dependence of reentry duration on wavelength was the same for electrical vs. structural remodeling. However, the dynamics during atrial reentry varied between electrical, structural, and combined electrical and structural remodeling in several ways, including: (i) with structural remodeling there were more occurrences of fragmented wavefronts and hence more filaments than during electrical remodeling; (ii) dominant waves anchored around different anatomical obstacles in electrical vs. structural remodeling; (iii) dominant waves were often not anchored in combined electrical and structural remodeling. We conclude that, in simulated atrial fibrillation, the wavelength dependence of reentry duration is similar for electrical and structural remodeling, despite major differences in overall dynamics, including maximal number of filaments, wave fragmentation, restitution properties, and whether dominant waves are anchored to anatomical obstacles or spiralling freely.

## Author Summary

Atrial fibrillation is an abnormal heart rhythm characterized by rapid and irregular activation of the upper chambers of the heart. Atrial fibrillation often shows a natural progression towards longer and more frequently occurring episodes and often occurs in patients with existing heart disease(s). Because atrial fibrillation has several variants, is complex in nature, and evolves over time, it is very difficult and expensive to study comprehensively in large-animal models, in part due to the inherent technical difficulties of imaging whole-atria electrophysiology in vivo. Predictive multiscale computational modeling has the potential to fill this research void. We have incorporated aspects of chronic atrial fibrillation to model some of its various disease states. As such, this study represents the first comprehensive computational study of chronic atrial fibrillation maintenance in a biophysically detailed cell model in a realistic three-dimensional anatomy. Our simulations show that disease-like modifications to cellular processes, as well as to the coupling between cells, perpetuate simulated atrial fibrillation by accelerating the rhythm and/or increasing the number of circulating activation waves. Given the model's ability to reproduce a number of clinically and experimentally important features, we believe that it presents a useful framework for future studies of atrial electrodynamics in response to, e.g., ion channel mutations and various drugs.

## Introduction

Atrial fibrillation (AF) is a cardiac arrhythmia characterized by rapid and irregular atrial activation. Such desynchronized activation may occur when multiple waves circulate the atria. Unlike ventricular fibrillation, where unsynchronized activation of the ventricles (the main pumping chambers of the heart) causes an immediate and typically fatal loss of blood pressure, atrial fibrillation may be a repetitive, even chronic, disease. In fact, AF is the most common sustained cardiac arrhythmia in the United States and the rest of the developed world [1], [2], with more than 2.3 million sufferers in the U.S. [3]. AF becomes increasingly common with age [2] and is associated with significant mortality and morbidity, such as heart failure and stroke [1].

AF is more prominent in the context of alterations in atrial tissue properties – due to disease, arrhythmias, or age – known as remodeling. In fact, AF itself leads to remodeling, causing electrophysiological (“electrical”), contractile, and structural changes [4]. Although AF can typically be reversed in its early stages, it becomes more difficult to eliminate over time due to such remodeling – hence the expression “AF begets AF” [5].

A central hypothesis for why AF begets AF is that electrical and structural remodeling due to chronic or persistent AF shorten the action potential wavelength, which measures the spatial extent of the action potential. Such wavelength shortening allows more waves to fit in the atria and maintain the arrhythmia [6]. Electrical remodeling primarily shortens the refractory period and the action potential duration (APD) of the atrial action potential, while structural remodeling impedes propagation and hence decreases conduction velocity (CV). Since the wavelength is given as the product of APD and CV (or, alternatively, the product of the effective refractory period and CV), electrical remodeling and structural remodeling both decrease the wavelength, thus potentially perpetuating AF. Additionally, the stability of reentrant waves may be affected by remodeling. Prior modeling work has shown that flattening APD restitution (the dependence of APD on the previous resting interval or Diastolic Interval, DI), which typically occurs as a consequence of electrical remodelling [7], may stabilize reentry [8]. Likewise, diffuse fibrosis, which may occur during structural remodelling [9] may stabilize reentrant waves [10].

Clinically, because electrical and structural remodeling typically present jointly in patients with chronic AF, their effects are difficult to separate. Animal models of primarily electrical remodeling (due to rapid atrial pacing) and predominantly structural remodeling (induced heart failure or mitral regurgitation) exist, however the rapid atrial pacing models also typically develop some degree of structural remodeling while the heart failure animals undergo some concomitant electrophysiological changes [9], [11]. We therefore decided to use computer modeling as a means to investigate the mechanisms of how APD shortening due to electrical remodelling, and CV slowing due to structural remodelling, influence the duration and spatiotemporal dynamics of simulated AF in a computational multiscale model of human electrophysiological dynamics and substrates. Because structural aspects of the complex atrial anatomy are important for, e.g., anchoring waves to anatomical obstacles and thus influencing the duration of reentrant activity, we use an anatomically detailed structural model of the human atria.

## Methods

### Cellular and anatomical models

We model the atria using the Courtemanche et al. cellular model of human atrial cell electrophysiology [12], with computational cells diffusively coupled to their nearest neighbors in an anatomically derived, three-dimensional structural model of the human atria [13]. As in previous work from our group [14], we increase the conductance of the inward rectifier current, I_K1_, here by 75%, in order to get the baseline action potential duration and resting membrane potential closer to experimentally observed values. Further, as in our previous work [14], we fix the intracellular concentrations of K^+^ and Na^+^ (at 139.0 mM and 11.2 mM, respectively) to avoid long-term drift.

The anatomical model incorporates heterogeneous coupling, resulting in different conduction velocities in different anatomical regions, in agreement with human data [13], [15]. Specifically, the model exhibits fast conduction in Bachmann's bundle, the pectinate muscle network, the crista terminalis, and the limbus of the fossa ovalis (120 cm/s during sinus pacing); slow conduction in the isthmus and the fossa ovalis (36 cm/s), while the remaining (bulk) atrial tissue has intermediate conduction velocity (65 cm/s). The different regions are shown in Fig. S1 in Text S1 (online Supporting Information). The model does not include anisotropy. In human atria, the bulk atrial muscle has a more random fiber orientation than the fast conduction pathways (and also more random than the ventricular myocardium), which have well-organized orientations along the bundles [13]. However, due to the strip-like anatomy of the fast tissues (Fig. S1 in Text S1), anisotropy is predicted to play a minor role there.

This mathematical representation of the atria reproduces basic features such as depolarization time and spatial profile during normal (sinus) pacing [13].

### Numerical methods and implementation

The three-dimensional anatomical model is discretized in a 300×285×210 grid of spatial nodes, with a spacing of *Δx* = 0.025 cm, and no-flux boundary conditions. The equations were solved using an operator-splitting method [16] with forward Euler integration of both operators. We used a fixed time step of *Δt* = 0.01 ms for the partial differential equation describing the diffusion of voltage. The cellular model was integrated using an adaptive time step [16].

The code was parallelized using OpenMP, and run on multi-core machines. Simulating 60 s of reentrant activity took 5 days on a 24-core machine (2.66 GHz Intel® Xeon® X7460 processors, 128 GB memory). Because the simulations are this computationally costly, simulations were stopped when reentry terminated or at 60 s (in which case, the arrhythmia was classified as sustained), whichever came first.

### Electrical and structural remodeling

Electrical remodeling due to chronic AF was simulated as in previous work, incorporating a 70% decrease in the conductance of the L-type calcium current (I_CaL_), a 50% decrease in the conductance of the transient outward current (I_to_), and a 50% decrease in the conductance of the atrial-specific, ultra-rapid potassium current (I_Kur_) [7]. These values are based on current recordings in cells isolated from human atrial appendages. We refer to this set of values as 100%, or full, electrical remodeling. In order to simulate different degrees of electrical remodeling (10–90%), in some simulations the percentage changes in the three affected conductances were downscaled by the same factor.

We simulate structural remodeling by decreasing the diffusion coefficients (i.e., the coupling strengths between computational cells), which reduces conduction velocity. The three different nominal diffusion constant values (assigned to fast, bulk, and slow conducting tissue) were scaled by the same factor. Maximal structural remodeling was set to a 50% decrease in diffusion, causing the time for full activation of the atria with sinus pacing to increase from 108 ms to 149 ms. However, as experimental and clinical data show a large range of conduction impairment with structural remodelling [9], we investigate two more levels of structural remodeling, using downscaling in diffusion of 70% and 83% (increasing activation time to 119 ms and 130 ms, respectively).

### Initiation of reentry

Because the duration of reentrant episodes may depend on where the reentry is initiated, we simulated reentry initiated at three different locations: the left atrial free wall, the left atrium near the left pulmonary veins, and the right atrial free wall. At each of these locations, reentry was initiated using a cross-gradient protocol, using a stimulus current of 80 nA/µF for 1 ms. Because the vulnerable window for reentry initiation is very small in the non-remodeled virtual tissue, we applied a brief hyperpolarizing clamp (−80 mV for 1 ms) to the region of the second wave excitation, 30 ms after its initiation. This allows for earlier reentry into this region and increases the vulnerable window.

The coupling interval (i.e., the time between the first and the second excitation) was varied systematically between simulations in steps of 10 ms within the vulnerable window. The size of the vulnerable window varies with variation in electrical and structural remodeling parameters, but was in the range of 30–60 ms, such that 4–7 reentry simulations were initiated at each location. Applied to three different locations, this means that for a given set of parameters describing the degree of electrical and/or structural remodeling, 12–21 simulations were run with different initiations.

### Analyses

DI and APD were recorded from 16 different locations, spread evenly throughout the atria (see Fig. S2 in Text S1). The APD was measured as the time from the crossing of −70 mV on the upstroke to the crossing of −70 mV during repolarization. Inversely, DI was measured as the time between the crossing of −70 mV during repolarization to the crossing of −70 mV on the next upstroke.

The wavelength (WL) is difficult to measure accurately during reentrant activity, even in computational studies [17], due to wave collisions and irregular wave propagation. We use a method similar to that employed by Graux et al. (Ref. [18]) and determine CV during periodic pacing in the left atrial free wall. However, rather than obtaining CV for very few pacing rates as necessitated in the clinic, we systematically varied the pacing rate to establish the dependence of the CV on diastolic interval. Such restitution curves were obtained for all the combinations of the different levels of electrical and structural remodeling simulated. We later used these restitution curves to estimate local CV for DI values measured during reentry and, finally, to compute the local wavelength as WL = APD×CV. Note that this definition of the wavelength is more practical than WL = ERP×CV, where ERP is the effective refractory period, since ERP measurement requires a series of stimuli and cannot be measured directly during simulated AF. In paced tissue simulations, we found that APD underestimates ERP by 9–16 ms (4–10%) depending on pacing rate and level of remodeling. Hence, our calculations of the wavelength using APD are presumably 4–10% larger than estimates based on ERP.

Atrial fibrillation and flutter can be characterized by the number of wavelets present in the tissue. As in our group's previous work [14], we compute the location of wave tips (filaments) from the crossing of two isopotential curves (the crossing of −30 mV on the upstroke), separated in time by 2 ms. The number of separate filaments is determined by applying a k-means clustering analysis to the filament location data. To characterize individual simulations we use the maximal number of filaments present in that run.

Dominant waves were defined as waves existing for at least five rotations. Their locations were determined directly from the filament location data or (in the case of anchored waves without filaments) based on periodicities in the transmembrane potential from the 16 recording sites, as well as visual inspection of isopotential surface maps.

## Results

### Electrical and structural remodeling increase reentry duration

As shown previously, electrical remodeling leads to shortening of the APD [7]. In particular, in our simulations of tissue strands, full electrical remodeling reduces the APD at 1 Hz pacing from 228 ms to 135 ms, while at 5 Hz the APD is shortened from 134 ms to 103 ms. This reduction in the amount of APD shortening with faster pacing demonstrates the flatter APD restitution occurring with electrical remodeling (see Fig. S3A in Text S1). The CV is unchanged with electrical remodeling (Fig. S3C in Text S1). In contrast, structural remodeling decreases CV (CV restitution slope remains largely unchanged; Fig. S3D in Text S1), while the APD is unchanged (Fig. S3B in Text S1).

As a measure of the effects of electrical and structural remodeling, we focus primarily on the duration of reentrant activity. In our three-dimensional model, in the absence of electrical and structural remodeling, reentrant activity is not sustained: in all simulations of normal tissue, with varying initiation time and location (see Methods), reentry ended 1–3 seconds after initiation. This is consistent with clinical findings in the normal human atria, where AF episodes typically self-terminate soon after initiation.

**Figure 1:**
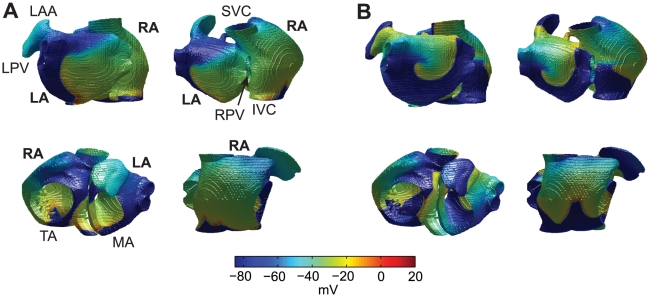
Reentrant activity in computer simulations of normal tissue (A) and tissue with full electrical plus structural remodeling (B). Different views are obtained at the same point in time for each simulation and show left atrial free wall (upper left), left and right atria in a posterior view (upper right), tricuspid annulus and mitral annulus (lower left), and right atrial free wall (lower right). Abbreviations: left atrium (LA), right atrium (RA), left pulmonary veins (LPV), right pulmonary veins (RPV), superior vena cava (SVC), inferior vena cava (IVC), tricuspid annulus (TA), mitral annulus (MA). Reentry was initiated in the left atrial free wall in both cases. Snapshots were taken after 510 ms (A) and 9 s (B).

Fig. 1A shows an example of non-sustained reentrant activity in normal tissue. The wavelength in this case is sufficiently long that the reentrant wave eventually runs into refractory tissue and dies out. In contrast, when simulating full electrical plus structural remodeling, reentry was sustained for 60 s in 18 of 21 simulations. With such electrical plus structural remodeling the wavelength is much shorter than in normal tissue (Fig. 1B), and the reentrant wave in the left atrial free wall does not self-terminate. Videos showing these dynamics with and without remodeling are available as Supporting Information (Videos S1 and S2).

**Figure 2:**
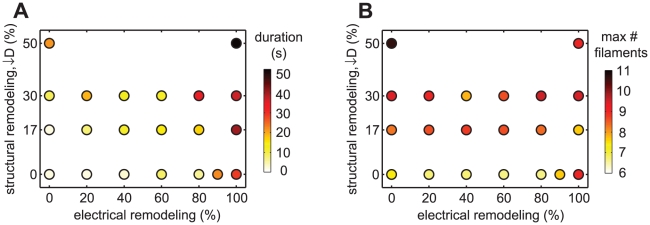
Remodeling increases reentry duration and maximal number of filaments. Dependence of mean duration of reentrant activity (A) and maximal number of filaments (B) on the levels of electrical and structural remodeling.

To investigate whether this maintained reentrant activity results from more waves being present versus those present being more stable, we measured the maximal number of filaments in a simulation, as well as the mean duration of reentrant activity, while varying the level of electrical and structural remodeling. Electrical and structural remodeling both lead to increases in the duration of reentrant activity, and the combination of electrical plus structural remodeling gives even longer sustained reentry (Fig. 2A). Structural remodeling also causes an increased number of filaments, while the level of electrical remodeling does not exhibit a clear correlation with the maximal number of filaments (Fig. 2B). Note that the number of filaments present at baseline is consistent with previous experimental and modeling studies [14], [19], [20].

### Reentry maintenance and wavelength

**Figure 3:**
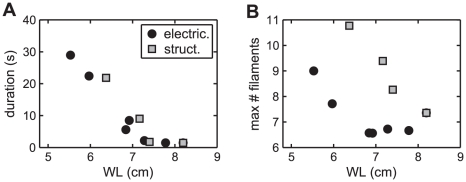
Shortened wavelength increases reentry duration and maximal number of filaments. Dependence of mean duration of reentrant activity (A) and maximal number of filaments (B) on the wavelength during electrical (black circles) or structural (gray squares) remodeling.

To test the hypothesis that a smaller wavelength perpetuates AF, we determined the dependence of the duration of reentrant activity on the estimated wavelength. For both electrical and structural remodeling, a decrease in WL is associated with longer reentry duration when WL is below a threshold value of around 7 cm (Fig. 3A). Interestingly, the dependence of reentry duration on WL is similar for both electrical and structural remodeling, suggesting that WL is a more important determinant of duration than other factors, such as APD restitution slope, that vary between electrical and structural remodeling.

In contrast, the dependence of the maximal number of filaments on WL is very different for electrical vs. structural remodeling, with more filaments present during structural than electrical remodeling for the same WL (Fig. 3B). This indicates that more conduction block and wavebreaks occur during structural remodeling, but might also be the result of fewer waves being anchored to anatomical obstacles, since an anchored wave does not necessarily have a filament. From analyzing the dynamics of the number of filaments, we found that with structural remodeling, frequent occurrences of conduction block and wavebreaks cause the larger maximal number of filaments relative to electrical remodeling. These wavebreaks often heal, such that the increase in filaments is transient (Figs. S4 and S5 in Text S1).

Taken together, these results demonstrate that the wavelength determines the duration of simulated AF, despite differences in dynamics such as APD restitution, conduction block, and number of filaments.

### Concomitant APD and CV variation

As mentioned above, in our simulations of periodically paced tissue strands, electrical remodeling shortens APD without changing CV, and structural remodeling decreases CV without affecting APD. If these dependencies hold during reentry in the three-dimensional atrial anatomy, then APD itself should be a marker for reentry duration during electrical remodeling, while CV should correlate with reentry duration during structural remodeling. Such markers might be valuable given the methodological difficulties in determining WL (see Methods).

**Figure 4:**
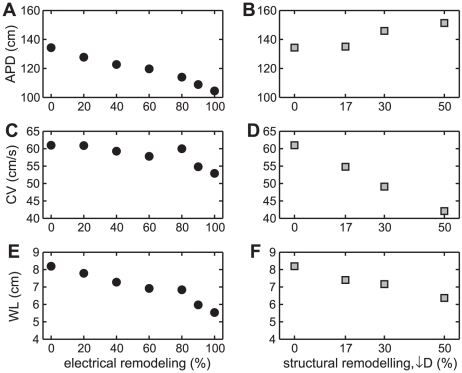
Primary and concomitant changes in APD and CV with remodelling. Dependence of APD (A and B), CV (C and D), and WL (E and F) on the degree of electrical or structural remodeling.

However, during reentry in the anatomical model with simulated electrical remodeling, there is both a decrease in APD and a concomitant fall in CV (Fig. 4A,C). For structural remodeling, there is a primary decrease in CV (Fig. 4D) and a secondary *increase* in APD (Fig. 4B). These results show first of all that APD alone (for electrical remodeling) and CV alone (for structural remodeling) are not accurate surrogates for WL during reentry. A similar finding was reported for AF/AFL inductance in a canine model [20].

Importantly, the secondary changes also suggest that the dynamically induced differences in the wave characteristics (APD and CV) may be due to differences in preferred pathways during reentry in the anatomical model, since differences in pathway lengths would affect the size of the excitable gap, and hence cause dynamical changes in DI, APD, and CV.

### Spatiotemporal organization of atrial reentrant patterns

Our different reentry initiation protocols allow a range of different spatiotemporal dynamics to occur. In all simulations that ran the full 60 s, the reentrant activity settled into a relatively periodic rhythm, with dominant waves remaining in a particular location (often circulating an anatomical obstacle). However, as shown in Table 1, the location of the dominant wave(s) varied significantly. In general, with only electrical remodeling dominant waves tended to be located in the left atrium, while dominant waves were found in the right atrium when we simulated structural remodeling only. With combined electrical and structural remodeling, dominant waves were in either or both atria.

**Table 1:** Anatomical organization of reentrant dynamics.

Electrical	(6)	Structural	(7)	Electrical+structural	(18)
PVs	(4)	TA (ccw)+IVC	(5)	LA free	(5)
TA (ccw)+SVC	(1)	TA (cw)+IVC	(2)	SVC	(3)
IA	(1)			LA free+SVC	(2)
				PVs	(4)
				RA free	(2)
				TA (cw)	(1)
				IA	(1)

Summary of spatiotemporal organization of sustained reentry with full electrical, full structural, and full electrical plus structural remodeling. *PVs* indicate pulmonary veins, *TA* tricuspid annulus, *IA* inter-atrial, *SVC* superior vena cava, *IVC* inferior vena cava, *LA free* left atrial free wall, *RA free* right atrial free wall, *cw* clockwise, and *ccw* counter-clockwise. Numbers in parentheses indicate numbers of occurrences.

**Figure 5:**
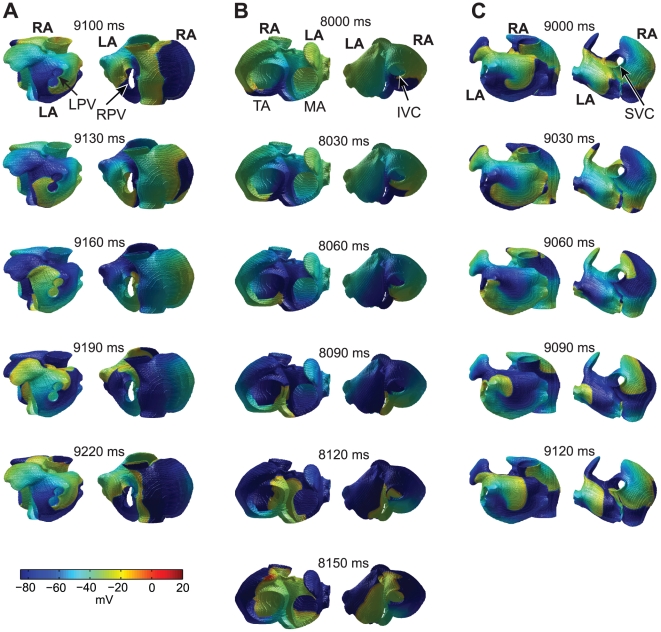
Typical long-term patterns of reentry in computer simulations of atrial tissue with different combinations of remodeling. Left and right columns in each of A, B and C, show two different views obtained at the same time points. Snapshots are 30 ms apart. Reentry anchored to the left pulmonary veins (LPV) during full electrical remodeling (A). Reentry around the tricuspid annulus (TA) and the inferior vena cava (IVC) during full structural remodeling (B). Un-anchored reentry in the left atrial free wall and anchored reentry around the superior vena cava (SVC) with full electrical plus structural remodeling (C).

More specifically, for electrical remodeling, 4 of 6 simulations resulted in reentry around the pulmonary veins (Table 1). Fig. 5A shows an example of such dynamics (see also Video S3 in the Supporting Information). A wave is anchored to the left pulmonary veins during the entire rotation (Fig. 5A, left). Another wave front is circulating the right pulmonary veins, but does not remain completely anchored for the entire rotation (Fig. 5A, right). Excitation spreads from the left atrium to the right.

During structural remodeling, all simulations of sustained reentry result in dominant waves rotating around the tricuspid annulus and the inferior vena cava (Table 1). In most cases (5 of 7), the rotation is counter-clockwise around the tricuspid annulus. An example is shown in Fig. 5B (and Video S4), with the wave anchored to the tricuspid annulus on the left, and the wave circulating the inferior vena cava on the right.

With electrical plus structural remodeling, the outcome is more varied. In some cases waves are anchored (to the pulmonary veins, the superior vena cava, or the tricuspid annulus). However, in some simulations, the dominant waves are un-anchored but spiral around the left or the right atrial free walls (Table 1). An example of such a scroll wave is shown in Fig. 5C, left (and Video S5). In this example, there is also a wave anchored around the superior vena cava in the right atrium (Fig. 5C, right, and Video S5), while in other simulations the excitation of the right atrium by the scroll wave in the left atrium is more irregular.

Note that the dominant periods vary with the different interventions. In the baseline (no remodeling) model, the mean period of (non-sustained) reentry is 176 ms. With full electrical remodeling, the shortening of the APD allows a faster mean rhythm of 137 ms. For full structural remodeling, the conduction slowing increases the main period to 205 ms. Interestingly, for full electrical plus structural remodeling, the period is the same as for only electrical remodeling (138 ms), suggesting that the preferred pathways are shorter on average for electrical plus structural remodeling. These values fall within the range seen in patients with paroxysmal and persistent AF [21], [22].

The different rhythms and their occurrence patterns (Table 1) correspond to clinical and experimental observations. The pulmonary veins frequently act as triggers of AF [23], and reentrant waves have been mapped in the pulmonary vein region [24], [25]. In canine models of structural remodeling, and in typical atrial flutter, excitation often occurs around the tricuspid annulus [26], sometimes in concert with reentry around the inferior vena cava [27].

## Discussion

We have incorporated aspects of electrophysiological and structural remodeling as a step in simulating the various disease states of AF. Our simulations show that electrical remodeling alone leads to rapid activation patterns, while structural remodeling causes wavebreaks and wave fragmentation. The decreases in wavelength due to remodeling makes different reentrant pathways possible and cause reentry perpetuation.

### Modeling atrial arrhythmias

Recent years have seen a large increase in modeling atrial-specific aspects of arrhythmogenesis. In particular, there has been increased development of anatomical models, powered by increasing computational speed and data handling (see, e.g., [28] for a review on modeling and [29] for technical details on computational and visualization aspects).

At the cellular level, multiple mathematical models describe the same ionic currents in different representations of human atrial myocytes. The Courtemanche et al. model [12] and the Nygren et al. model [30] are both well-established and have been compared in great detail [31], [32]. Although their simulated behavior can be quite different, we believe that neither is empirically better. Rather they may represent intrinsic variability. Given that neither is obviously better, we have opted to use the Courtemanche et al. model, largely because it is more widely used. The Nygren et al. model was recently updated in terms of some of its potassium currents [33] and its intracellular calcium handling system [34].

The effects of electrical remodeling on action potential morphology, in particular the role of the individual currents involved in remodeling [7], [35], have been studied at the cellular level. In two-dimensional tissue, electrical remodeling accelerates spiral waves generated with both the Courtemanche et al. model [32], [36] and the Nygren et al. model [32]. With electrical remodeling there is also a decrease in spiral wave meandering in the Courtemanche et al. model [32], [36], but not with the Nygren et al. model [32]. Such a decrease in spiral meandering with the Courtemanche et al. model is enhanced with increased I_K1_ [37] and can indeed occur in simulated atrial tissue with increased I_K1_ in the absence of electrical remodeling [38]. Increased I_K1_ also accelerates spiral waves [37], as does another inwardly rectifying current I_K,ACh_, which is triggered in cholinergic AF [39], [40].

In simulated tissue (Ref. [36], as well as in our simulations (Fig. 4)), electrical remodeling decreases the wavelength in tissue strands and causes arrhythmias in anatomical models to be of longer duration (Fig. 2; [32], [36]). Decreasing the calcium current conductance, which is a main component of electrical remodeling, has similar effects in a human anatomical structure of virtual guinea pig ventricular cells [17], [19]. Combining electrical remodeling and left atrial dilation leads to increased vulnerability to reentry in an anatomical model [41], while combining electrical remodeling and decreased intercellular coupling causes shortened wavelength and sustained spiral wave activity in two-dimensional tissue simulations [42], consistent with our results.

Other lines of study, pursued with complex atrial models, include the effects of myocardial stretch on conduction [43], [44], incorporation of intrinsic APD heterogeneity [45], [46], [47], and simulated ablation [48], [49].

### AF mechanism

The mechanism underlying AF maintenance is not entirely clear. There are two predominant theories: (i) the multiple wavelet hypothesis and (ii) the “mother rotor” hypothesis. The multiple wavelet theory [50] hypothesizes that AF is composed of multiple interacting electrical wavelets and is maintained by the processes of wavebreak and reentry. The mother rotor theory [51] hypothesizes that, rather than the multiple, equally important wandering wavelets of the multiple wavelet hypothesis, there is one dominant “mother” reentrant wave that sheds and initiates daughter waves as conduction block occurs at multiple sites away from its core.

The dynamics in our simulations are characterized by one or two reentrant waves, rotating fairly periodically around an anatomical obstacle or un-anchored in the (left or right) atrial free wall. Hence, our simulations are more consistent with the mother rotor theory. Further, in the presence of simulated structural remodeling, waves emanating from these dominant waves often exhibit local conduction block and transient fragmentation. Recent high-density mapping studies of patients with persistent AF and structural heart disease also found increased occurrence of conduction block in the right atrium compared to patients without persistent AF and structural heart disease [52]. However, the AF dynamics in the former patient group is characterized by more epicardial breakthroughs and more waves than our simulations, possibly due to increased decoupling of neighboring myocyte bundles [52], [53]. Using non-invasive imaging techniques, Cuculich et al. determined the number of wavelets in AF patients as 1–5, with more wavelets in patients with long-term persistent AF (average 2.6) than in patients with paroxysmal AF (average 1.1) [25], consistent with our findings. However, these patients also had a considerable number of focal sites (possibly due to spontaneous triggering, microreentry, or epicardial breakthroughs) [25], not seen in our simulations.

Atrial flutter (AFl) is usually associated with a single macroreentrant circuit and may be very regular. However, AF can also exhibit a large degree of both temporal periodicity and spatial organization [54], [55]. Hence, our simulated arrhythmias demonstrate characteristics of both AF and AFl. Clinically, AF and AFl may occur in the same patient, and AFl often converts to AF.

### Spatiotemporal organization of reentry

By initiating reentry at different locations and at different times, we were able to explore a large range of spatiotemporal dynamics in our simulations. A large variety of ensuing dynamics is also observed clinically and experimentally. While much of this variability may stem from differences among experimental AF models and patient-to-patient variation in underlying heart disease, age, and stage of remodeling, there is considerable variability in activation patterns even among patients with the same underlying condition [22].

Importantly, many features observed in our simulations correlate directly with experimental and clinical characteristics. One example is reentry around the pulmonary veins, seen in our model with electrical remodeling. Another example is reentry around the tricuspid annulus, with 5 of 7 simulations of sustained activity with structural remodeling resulting in the direction of rotation being counter-clockwise. Such right-atrial reentry, involving slow conduction through the isthmus, is typical in patients with AFl [26], [56] often with the majority (19/26) of cases having counter-clockwise rotation around the tricuspid annulus [57].

Further, with structural remodeling in our simulations, waves were also anchored around the inferior vena cava. Such dual-loop reentry involving the tricuspid annulus and the inferior vena cava is often observed in typical atrial flutter [27]. In addition to anatomical reentry, we also observed several examples of meandering scroll waves, which have been mapped experimentally and simulated computationally [58]. Finally we observed incidences of double-wave reentry, also observed clinically [56] and experimentally [59].

Rate gradients, with higher activation frequencies in the left atrium, exist in some animal models of AF [11], [54], [55] and have been observed in patients with both paroxysmal and persistent AF [22], [60], [61], [62]; however, other chronic AF patients do not exhibit such inter-atrial variability [21]. In our simulations of remodeled tissue, there are no significant left-to-right gradients in activation frequency. However, our simulated AF with electrical remodeling alone was primarily a left atrial phenomenon in the sense that most dominant waves were found in the left atrium. This suggests that under some circumstances, the left atrium may be the driver of AF even in the absence of electrophysiological left-to-right heterogeneity (see below).

### Wavelength dependence of AF perpetuation

The extent to which perpetuation of AF depends on the wavelength varies considerably among different studies using different AF models and different methods for wavelength estimation. While several studies, both computational and clinical, have demonstrated a facilitation of AF maintenance with a decrease in wavelength [17], [18], others have not found such a dependence [63].

We found a clear functional dependence of reentry duration on wavelength in our simulations, and found that the wavelength must be below a value of around 7 cm for reentry perpetuation. However, the wavelength has to be considerably shorter for the majority of simulations to be sustained; this critical value for sustenance is about 5 cm. This value is consistent with a previous report of 5 cm using a direct measurement of the wavelength during simulated AF [17]. In contrast, clinical estimates for this threshold tend to be larger, with reported values of 12–13 cm [20], [64]. Importantly, as detailed in Ref. [17], these values are obtained through indirect methods, due to the inherent problem of insufficient spatial mapping, and tend to overestimate the wavelength.

### Electrical and structural remodeling in chronic AF

AF and AFl become increasingly common with age, and are also particularly frequent in patients with existing structural heart disease, valvular heart disease, coronary artery disease, ischemic heart disease, hypertension, or a history of heart attacks. Indeed, the majority of AF patients have one or more cardiovascular diseases in addition to AF. Electrical and structural remodeling due to chronic AF occur in concert with substrate changes due to any existing condition(s).

Changes in ionic currents due to chronic AF have been observed consistently in experimental models and in patients [9]. Recent studies have shown differences in several of the outward potassium currents between the left and the right atrial appendages, with some current differences being present in sinus rhythm or paroxysmal AF and others in chronic AF [65], [66]. As it is presently unclear what type of inter-atrial gradients these appendage differences represent, we did not incorporate any left vs. right electrical heterogeneity in our model at this point.

Structural remodeling in chronic AF shows more variability among patients and animal models than does electrical remodeling. Structural remodeling processes also occur on a much slower time scale than electrical remodeling, which may contribute to the substrate variability. The processes include myocyte hypertrophy, fibrosis, and changes in the expression levels of connexin, the protein comprising the gap junctions that couple cells. Fibrosis can manifest in both patchy patterns and diffuse morphologies. Fibrosis and decreases in connexin levels both impede propagation, while atrial dilation and cell hypertrophy increase atrial activation times. Our simulations using decreased coupling between virtual cells, which decrease conduction velocity and increase activation times, represent an unspecified propagation impairment that may be thought of as a decrease in connexin levels or a diffuse fibrosis.

### Limitations

Although contractile remodeling is associated with chronic AF, in addition to electrical and structural remodeling, we do not include such remodeling here. The main reason is that the current data on contractile remodeling in the human atria is insufficient to incorporate into a quantitative model. Further, attempts at including contractile remodeling into the cellular model would almost certainly require a considerable expansion of the intracellular calcium handling system such as that recently developed by Koivumäki et al. [34]. Such an expansion would significantly increase the computational load of the simulations, and as we are already operating on the cusp of computational tractability, we did not incorporate those changes in this study.

The pulmonary vein plays an important role in triggering AF and electrical isolation (ablation) of this area can be an efficient treatment for AF [23]. However, the pro-arrhythmic role of the pulmonary vein region is significantly lessened in chronic AF [61], [67], [68], which is also exemplified by the decreased success of ablation therapy in this patient group. The pulmonary vein region has electrophysiological properties that are different from other regions of the atria, typically showing shorter refractory periods [67], [69]. The details of which ionic currents are responsible for this heterogeneity and how the electrophysiology changes with progression of AF are not known, and hence we did not attempt to include special heterogeneity of the pulmonary vein region in this work. Further, this study focused on the maintenance, rather than the initiation of AF; further studies will be required to help elucidate triggering of AF due to mechanisms such as repolarization alternans and ectopy [14], [70] in remodeled and healthy tissue.

As described in the Methods, the three-dimensional atrial structure includes a gross coupling heterogeneity, with fast, normal, and slow tissues. However, the model does not include anisotropy. More prominent anisotropy during some types of structural remodelling [9] may play a role in AF perpetuation.

We simulated structural remodeling as a decrease in coupling. A first step towards more realistic simulations of fibrosis could include implementing structural remodeling by randomly assigning a variable fraction of cells to be electrically inactive and surrounded by no-flux boundary conditions [10]. Test simulations show that such implementation of structural remodeling does not alter our conclusions. Recent studies have shown how fibrosis may affect repolarization [71] in addition to slowing down conduction - we did not include such effects in this work.

### Conclusions

Electrical and structural remodeling both shorten the atrial wavelength; electrical remodeling primarily through a decrease in APD, while structural remodeling primarily slows down conduction. The wavelength determines the mean reentry duration in the same manner for electrical and structural remodeling despite major differences in overall dynamics (maximal number of filaments, conduction block, wave fragmentation, restitution properties, preferred dominant wave pathways, and whether dominant waves are anchored to anatomical obstacles or spiralling freely). As such, these findings have implications for our understanding of the mechanisms by which AF remodeling processes perpetuate AF.

## Supporting Information

Text S1 **Supplemental figures S1, S2, S3, S4, S5, S6 with legends.**
(PDF)

Video S1 **Short-lived reentry in non-remodeled atria (supplements** **Fig. 1A** **in the manuscript).** The different subfigures show different views: left atrial free wall (upper left), left and right atria in posterior view (upper right), tricuspid annulus and mitral annulus (lower left), and right atrial free wall (lower right).
(AVI)

Video S2 **Sustained reentry with combined electrical and structural remodeling (supplements** **Fig. 1B** **in the manuscript).** Same views as for Movie 1. The movie shows one rotation of the dominant waves.
(AVI)

Video S3 **Sustained reentry around the left pulmonary veins with electrical remodeling (supplements** **Fig. 5A** **in the manuscript).** The movie shows one rotation of the anchored wave.
(AVI)

Video S4 **Sustained reentry around the tricuspid annulus and the inferior vena cava during structural remodeling (supplements** **Fig. 5B** **in the manuscript).** The movie shows a rotation of the anchored waves.
(AVI)

Video S5 **Sustained reentry with electrical plus structural remodeling (supplements** **Fig. 5C** **in the manuscript).** There is an un-anchored wave in the left atrium and anchored reentry around the superior vena cava. One rotation of the dominant waves shown.
(AVI)
