# Supplementary material for: Effects of Electrical and Structural Remodeling on Atrial Fibrillation Maintenance: A Simulation Study
Source: PLoS Comput Biol. 2012 Feb 23;8(2):e1002390. doi: 10.1371/journal.pcbi.1002390 (PMC3285569; doi:10.1371/journal.pcbi.1002390)
Supplement: Text S1 — Supplemental figures S1, S2, S3, S4, S5, S6 with legends. (PDF) [file pcbi.1002390.s001.pdf]

Text S1

Supplemental Figures and Legends

for

Effects of Electrical and Structural Remodeling on Atrial Fibrillation Maintenance: A Simulation Study

Trine Krogh-Madsen, Geoffrey W. Abbott, and David J. Christini

Abbreviations: left atrium, LA; right atrium, RA; left pulmonary veins, LPV; right pulmonary veins, RPV; Bachmann's bundle, BB; left atrial appendage, LAA; superior vena cava, SVC; inferior vena cava, IVC; tricuspid annulus, TA; mitral annulus, MA.

FIGURE S1

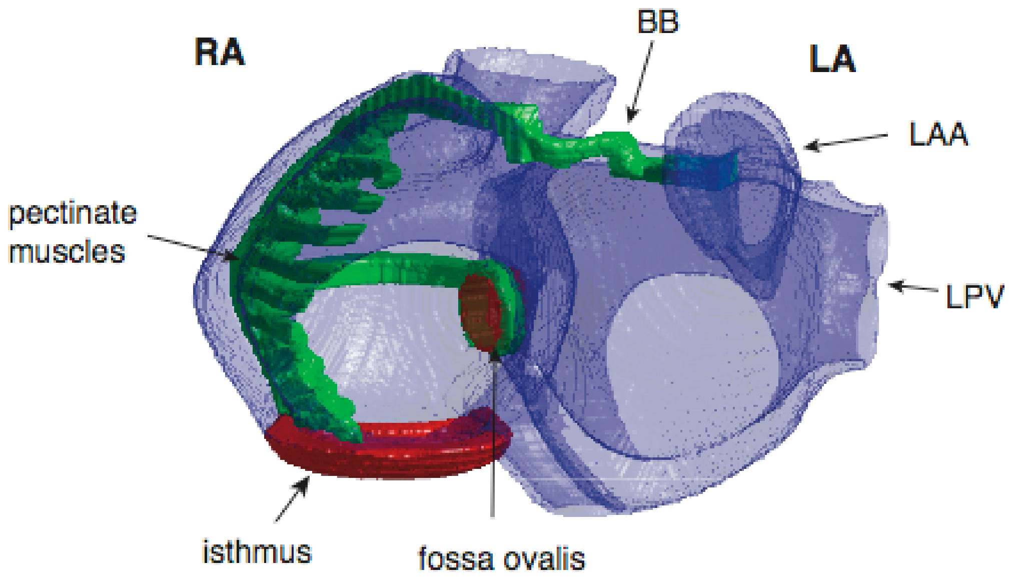

Figure S1 **Anatomical model of the human atria due to Harrild and Henriquez** (Ref. 12 in the manuscript). The right atrium measures 4.6 cm (anterior to posterior) by 4.2 cm (septal to lateral) by 4.5 cm (inferior to superior) and has a thickness of 0.21-0.29 cm. The left atrium measures 4.0 cm (anterior to posterior) by 4.0 cm (septal to lateral) by 4.1 cm (inferior to superior) with a thickness of 0.30-0.55 cm. The thickness of the left atrial appendage is considerably thinner (~1 mm). The structure is color-coded for the value of the diffusion constant with the largest value (6.0 cm^2/s; corresponding to the faster conduction) in Bachmann's bundle, the pectinate muscle network, the crista terminalis, and the limbus of the fossa ovalis (green). The smallest value (0.75 cm^2/s) gives slow conduction in the isthmus and the fossa ovalis (red). The remaining atrial tissue (blue) has an intermediate value of the diffusion constant (2.0 cm^2/s). Anterior view.

FIGURE S2

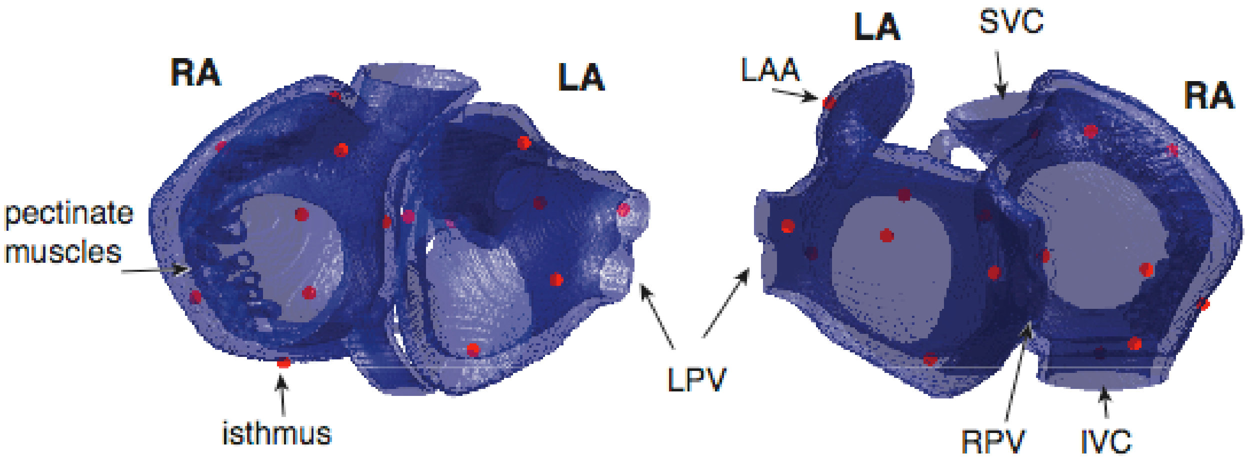

Figure S2 **Location of the 16 recording sites for action potential duration (APD) and diastolic interval (DI).** There are seven sites in each atrium and two interatrial locations. Left: anterior (left) and posterior views (right).

FIGURE S3

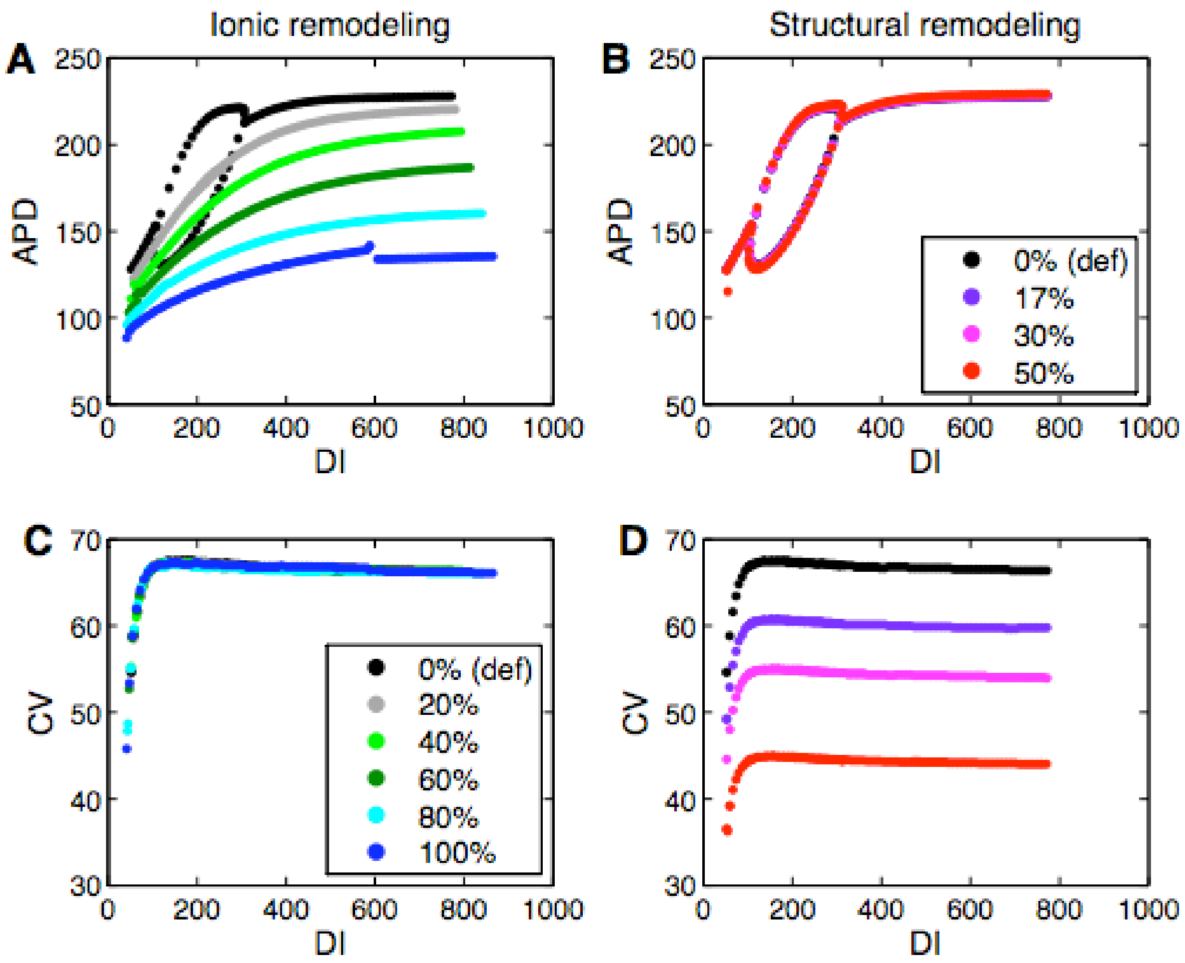

Figure S3 APD and conduction velocity (CV) restitution curves. Data points are from simulations in a 1-dimensional cable using a dynamic (S1S1) stimulation protocol.

FIGURE S4

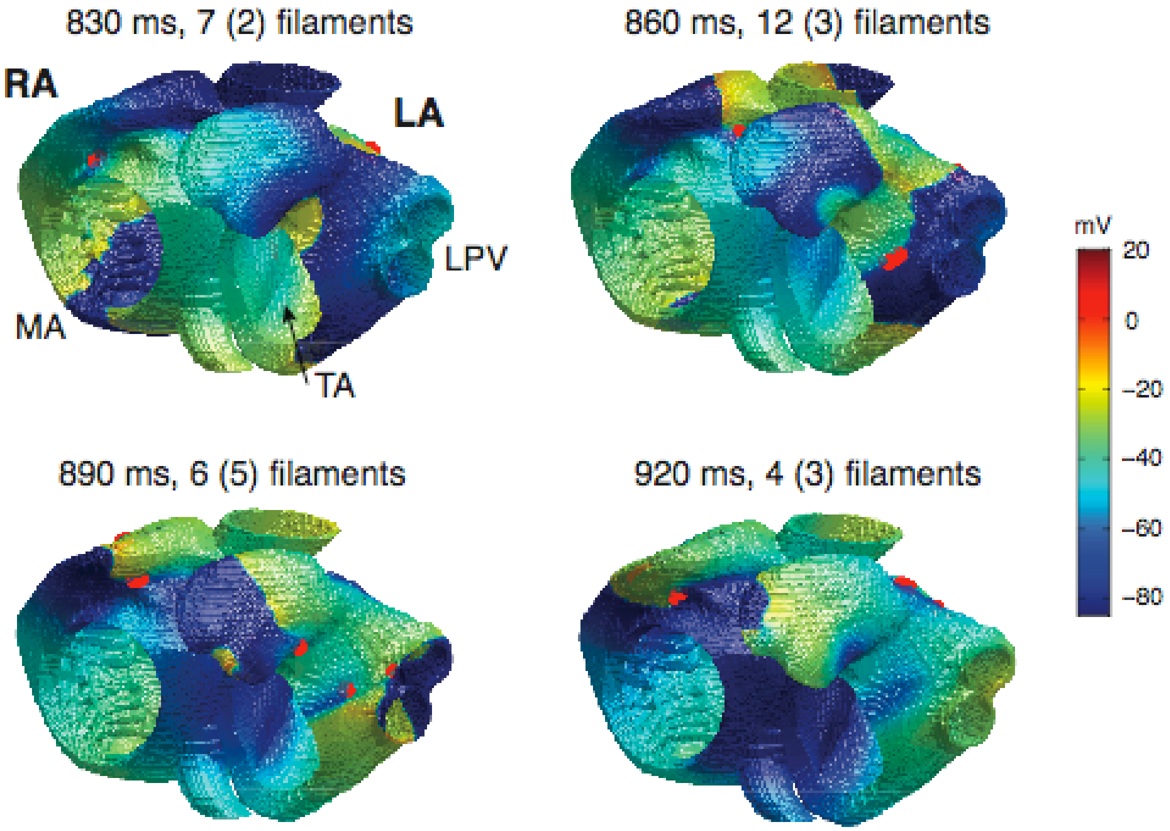

Figure S4 Transient increase in filaments during reentrant activity with structural remodeling. Locations of filaments are indicated by red dots. Snapshots are 30 ms apart. Numbers (numbers in brackets) indicate the number of filaments present (visible) at each time. Anterior view with mitral and tricuspid annuli.

FIGURE S5

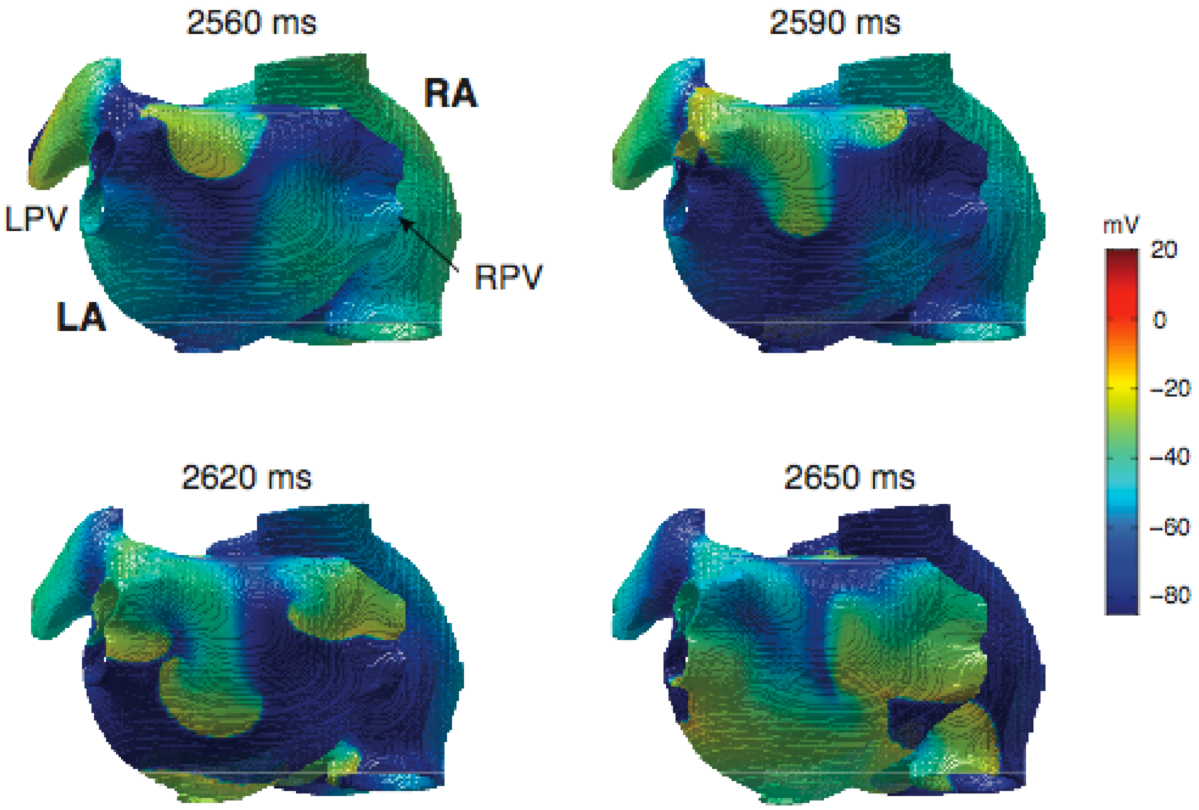

Figure S5 **Wave breakup during reentrant activity with structural remodeling.** Snapshots are 30ms apart. Posterior view of left atrium.

FIGURE S6

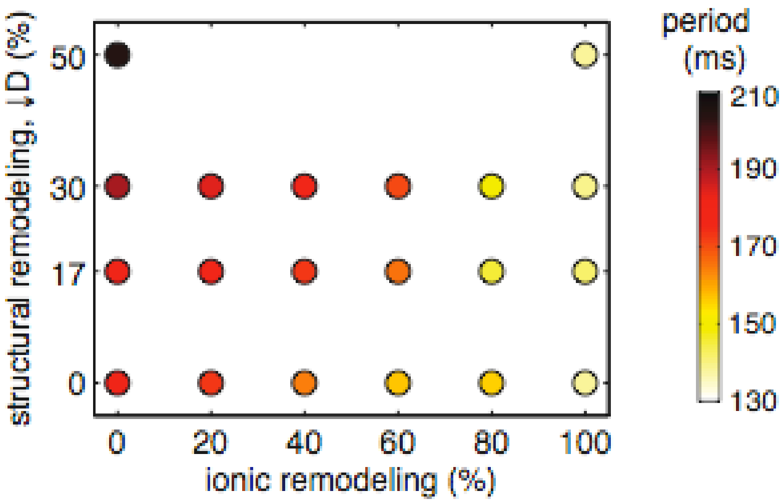

Figure S6 Average period of reentrant activity in atrial structure with different levels of electrical and structural remodeling. Electrical remodeling decreases the average period, while structural remodeling increases it.
